# Using a Natural Language Processing Approach to Support Rapid Knowledge Acquisition

**DOI:** 10.2196/53516

**Published:** 2024-01-30

**Authors:** Taneya Y Koonce, Dario A Giuse, Annette M Williams, Mallory N Blasingame, Poppy A Krump, Jing Su, Nunzia B Giuse

**Affiliations:** 1 Center for Knowledge Management Vanderbilt University Medical Center Nashville, TN United States; 2 Department of Biomedical Informatics Vanderbilt University School of Medicine Vanderbilt University Medical Center Nashville, TN United States

**Keywords:** natural language processing, electronic health records, machine learning, data mining, knowledge management, NLP

## Abstract

Implementing artificial intelligence to extract insights from large, real-world clinical data sets can supplement and enhance knowledge management efforts for health sciences research and clinical care. At Vanderbilt University Medical Center (VUMC), the in-house developed Word Cloud natural language processing system extracts coded concepts from patient records in VUMC’s electronic health record repository using the Unified Medical Language System terminology. Through this process, the Word Cloud extracts the most prominent concepts found in the clinical documentation of a specific patient or population. The Word Cloud provides added value for clinical care decision-making and research. This viewpoint paper describes a use case for how the VUMC Center for Knowledge Management leverages the condition-disease associations represented by the Word Cloud to aid in the knowledge generation needed to inform the interpretation of phenome-wide association studies.

## Introduction

The rapid advancement and availability of artificial intelligence (AI) approaches provide biomedical informatics groups with opportunities for exploring and generating insights from internal and external data at scale to enhance health sciences research and clinical care [1,2]. One such opportunity is using natural language processing (NLP) to extract usable knowledge from the vast amounts of structured and unstructured clinical data captured daily via the electronic health record (EHR). Insights from this process can be used to inform patient care, target information provision, and generate research hypotheses. This paper presents some of the activities that such usable knowledge makes possible.

Vanderbilt University Medical Center (VUMC) maintains an electronic health repository containing data for over 4.6 million individuals, going back to 1995, which includes structured data (eg, laboratory results and vital signs), textual data (eg, provider notes and radiology interpretations), reports (eg, electrocardiograms and pulmonary function test results), and image data. Included in this vendor-agnostic repository are all VUMC patient data captured from the in-house developed StarPanel EHR (VUMC) dating back to 2001 [3] and VUMC’s current vendor-based EHR (Epic; Epic Systems Corporation), which was implemented in 2017 [4]. Roughly 850,000 new documents are added daily.

To identify and quickly represent the most critical information about a particular patient or population from this large data set, VUMC established the Word Cloud, a real-time and at-scale concept extraction tool that uses NLP to create a visual, time-oriented representation of clinical data [5-7]. The Word Cloud NLP uses a rules-based, finite-state machine approach to process all nonimage incoming documents in real time and extract coded concepts using the Unified Medical Language System (UMLS) terminology [8]. With a processing speed of more than 50,000 documents per minute, the Word Cloud NLP is faster than currently available concept extraction NLP tools such as Apache cTakes (50,000 documents per hour; Apache Software Foundation, Mayo Clinic) [9] and MetaMap (22 citations per minute; National Library of Medicine) [10]. The rapid speed allows for better integration into the clinical workflow as real time–generated Word Cloud concepts are immediately presented to health care providers as they access the feature in the medical chart. The system handles all linguistic phenomena in clinical text, including acronyms, abbreviations, misspellings, negation, family history, uncertainty, and differential diagnosis. Excluding image data, the entire EHR repository is included in the Word Cloud NLP database, which uses close to 14,000 UMLS concepts to index 1.7 billion documents. In addition to the individual patient concepts, which include pointers to the original documents, the database also includes population-level associations of any pair of concepts.

The original purpose of the Word Cloud data was to provide a user interface that displays all concepts extracted from a patient’s clinical documents in a word cloud display, with the size of each concept indicating how often the concept was documented for the patient. This interface is available to all users of the EHR. The Word Cloud data have been used since 2019 to generate clinical alerts for a variety of situations, such as flagging patients with implanted cardiac devices and a positive blood culture, patients with signs of serious inflammation due to immune checkpoint inhibitors, or patients with Andersen-Tawil syndrome who might be candidates for enrollment into a research study. The Word Cloud data drive real-time decision support by injecting detected concepts back into the VUMC EHR [11]. Because all the concepts extracted by the Word Cloud NLP are stored in the enterprise data lake, these data are also available for retrospective research and can be easily combined with other data such as the International Statistical Classification of Diseases codes or coded medications data [11].

The Center for Knowledge Management (CKM) has explored how the Word Cloud can be leveraged by information scientists engaged in EHR projects. The CKM facilitates the discovery and integration of external knowledge into medical practice and promotes curation, archiving, and reuse of internal knowledge across VUMC [12-15]. This viewpoint paper details how the CKM’s innovative application of the Word Cloud enhances knowledge generation processes and describes future directions for NLP in knowledge management.

## Case Description

Collaborations with medical center researchers comprise the majority of the CKM’s partnership activities. A recent project to inform the interpretation of phenome-wide association studies (PheWASs) using evidence-linked knowledge bases illustrates these types of partnerships [16]. PheWASs examine relationships between markers (genetic or nongenetic) and phenotypes, producing extensive lists of possibly relevant marker-phenotype associations [17,18]. A methodological approach to compare known associations with PheWAS results can make it easier to identify potentially novel PheWAS outcomes [16]. Knowledge bases—created in part from synthesized evidence sources and primary literature documenting disease causes, risks, and complications—can be used for these comparisons.

For this research collaboration, the CKM created a “condition flowchart” with the causes, risk factors, and complications of a given medical condition. The sources consulted to create the flowchart include evidence synthesis resources (eg, UpToDate; UpToDate, Inc), medical textbooks (eg, Goldman-Cecil Medicine), and consumer health websites (eg, MedlinePlus; National Library of Medicine). From each source, the CKM team identified all causes, risk factors, and complications for the condition of interest and added them to the flowchart. Our collaborators then used the flowchart to create a knowledge base of phecodes for the PheWAS analysis. During flowchart creation, the CKM leveraged the Word Cloud to identify meaningful disease-condition associations—based on real-world population-level data—and target appropriate primary literature to substantiate the observed linkages.

## Identifying Meaningful Condition Associations From the EHR

Each flowchart focuses on a specific clinical condition (eg, hypertension and hypotension), which is searched against the Word Cloud. Using a population-level analysis feature, the Word Cloud returns a list of all UMLS concepts represented in the EHR records of patients with the specified condition. The expected value is calculated for each UMLS concept [19] and the ratio of actual-to-expected patient cases (ie, strength of association) is then used to rank the list of causes, risk factors, and complications on the flowchart. This ranking thus provides our team with rapid knowledge acquisition of what is associated with the condition of interest. The actual-to-expected ratio for concept 1 and concept 2 is computed as follows:



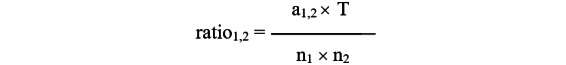



where T=total population size, a_1,2_=number of patients with both concept 1 and concept 2, n_1_=number of patients with concept 1, and n_2_=number of patients with concept 2.

**Figure 1:**
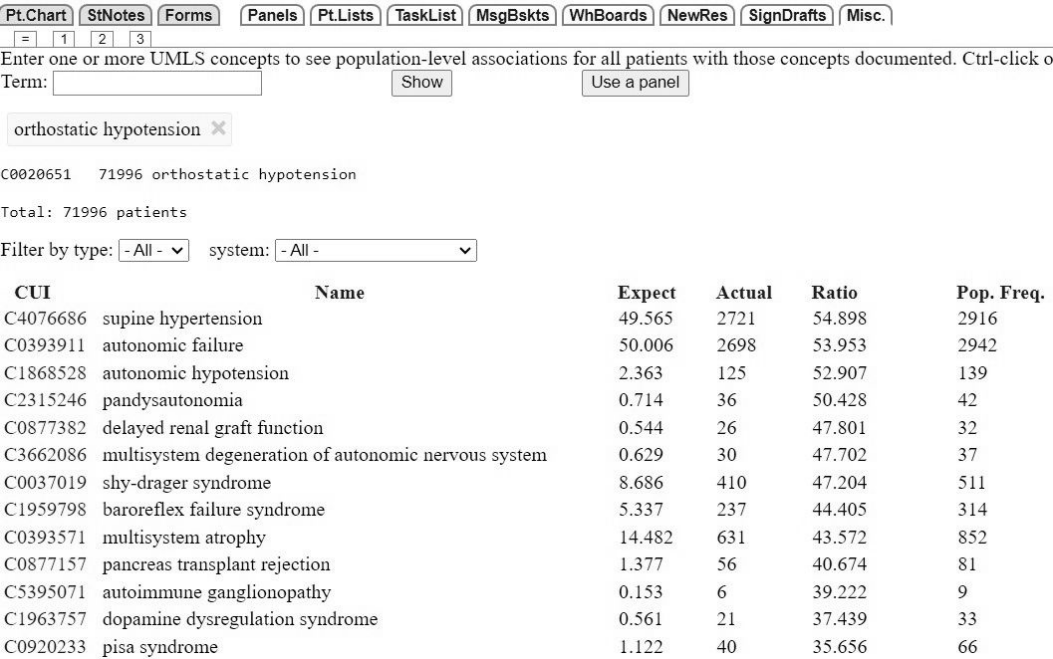
Snapshot of the Word Cloud population-level list of UMLS concepts associated with orthostatic hypotension. Concepts are listed in descending order by the strength-of-association ratio, that is, the ratio of actual to expected number of cases in the VUMC EHR with the pairwise association of UMLS concepts. The population frequency of each term is also displayed. The ratio is used to rank the condition flowchart. CUI: concept unique identifier; EHR: electronic health record; Misc.: miscellaneous; MsgBskts: message baskets; NewRes: new results; Pop. Freq.: population frequency; Pt.Chart: patient chart; Pt.Lists: patient lists; StNotes: Star Notes; UMLS: Unified Medical Language System; VUMC: Vanderbilt University Medical Center; WhBoards: white boards.

A strength of association ratio of 15 or higher indicates that the concept occurs more often than expected by chance and signifies a meaningful relationship between the UMLS concept and the condition. Figure 1 provides an example of the UMLS concepts most associated with orthostatic hypotension in 71,996 patients.

The Word Cloud also aids in identifying concepts most applicable to guide the ranking by displaying each term’s UMLS semantic type. In the UMLS Metathesaurus, each concept term is assigned to 1 or more of 127 types in the vocabulary’s hierarchical semantic network [20]. Semantic types most relevant for comparison with the condition flowchart include disease or syndrome, injury or poisoning, mental or behavioral dysfunction, sign or symptom, finding, and congenital abnormality. The Word Cloud provides a filter to exclude concepts with semantic types nonrelevant to this task (eg, procedures).

The Word Cloud often lists multiple UMLS concepts that can be grouped to correspond with a single term on the condition flowchart. For example, the Word Cloud concepts associated with orthostatic hypotension include Shy-Drager syndrome, multisystem degeneration of autonomic nervous system, and multisystem atrophy (Figure 1). In 1998, Shy-Drager syndrome was newly categorized as a multisystem atrophy and is no longer the preferred term [21]; the UMLS also lists it as a narrower concept of the term “multiple system atrophy” [8]. In the UMLS, the relationship between “multiple system atrophy” and “multisystem degeneration of autonomic nervous system” is vaguely and imprecisely defined as an “RO” relationship type. RO relationships are described as “other than synonymous, narrower, or broader,” however, in this case, the RO determination in the UMLS lacks the relationship attribute that is normally included [8]. The phecodeX map, the term mapping table used for the CKM collaborator’s PheWAS, matches “multisystem atrophy” to the phecode “multi-system degeneration of the autonomic nervous system” [22]. Given the evolution of the Shy-Drager syndrome terminology, the UMLS, and the phecodeX mapping, we subsequently considered all 3 of the Word Cloud concepts as a group of related terms; the highest ratio within the group was then used to rank order the condition flowchart.

Through the combined processes of documenting actual-to-expected case ratios of the Word Cloud’s relevant UMLS concepts, excluding nonrelevant semantic types, and grouping related concepts, our team creates rank-ordered lists of disease causes, risk factors, and complications reflecting our medical center’s real-world clinical data.

## Substantiating Disease-Condition Associations With Evidence

**Figure 2:**
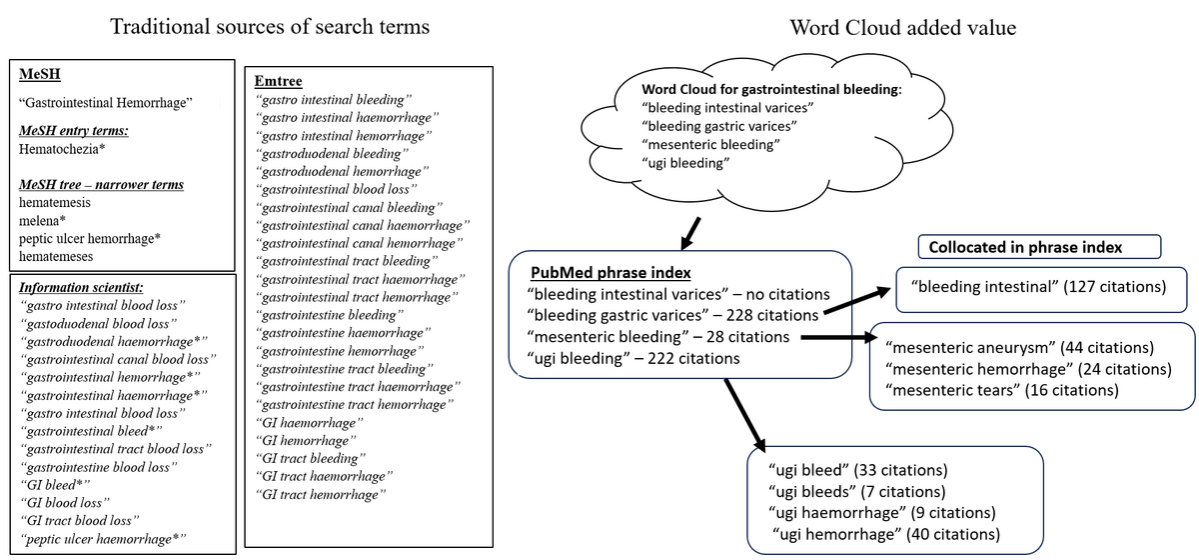
Word Cloud concepts leading to supplemental terminology for a search strategy on gastrointestinal hemorrhage. An asterisk denotes the truncation of a term or phrase to capture permutations. GI: gastrointestinal; MeSH: Medical Subject Headings; UGI: upper gastrointestinal.

Providing primary literature to substantiate the associations on the condition flowchart is a key component of our research collaboration. CKM information scientists derive synonyms from the Word Cloud to strengthen the search strategy. When conducting a search, they first compile controlled vocabulary and synonyms from Medical Subject Headings and Emtree [23,24]. Next, they brainstorm additional permutations and extract terms from a scan of the literature; these keywords are subsequently checked for inclusion in the PubMed phrase index. Figure 2 shows an example of this process for the UMLS concept “gastrointestinal bleeding.” Consulting the Word Cloud identified 4 phrases that were not in the initial list of search strategy terms; 3 were found in the PubMed phrase index. Additional terminology was found by scanning collocated terms in the phrase index.

In addition to aiding with identifying terminology and concepts to build upon our search strategies, we increasingly realize the importance of the Word Cloud’s actual-to-expected patient case ratio for locating appropriate evidence. When creating the condition flowchart, our team may encounter associations for which it is difficult to locate substantiating evidence. In these cases, a Word Cloud ratio that is nonexistent, or lower than 15, can aid in validating literature scarcity. For example, snake bites can lead to nonseptic distributive shock [25]. In the Word Cloud, the association between snake bite and distributive shock has a low ratio of 5.38. Substantiating evidence for the association was found only in case reports and case series (ie, studies with few patients). Similarly, searching for literature to support hypertrophic cardiomyopathy as a cause of obstructive shock yielded only case reports as the best available evidence. A review of the Word Cloud UMLS concepts revealed a ratio of 8.7. In these instances, the evidence may still be used, but the low ranking, due to the low ratio, aids in understanding the strength of association when compared with other causes, risk factors, and complications listed on the condition flowchart.

## Conclusions

This viewpoint paper describes a novel use of an institution’s AI-driven, large-scale aggregation of condition-specific patient data extracted from free-text clinical documents. The Word Cloud NLP system can inform and guide knowledge generation processes by enhancing our ability to represent, substantiate, and prioritize condition associations for use in PheWAS interpretation.

The VUMC Word Cloud NLP is a valuable resource that provides real-time concept extraction from all clinical documentation and makes the resulting data viewable interactively, available for real-time decision support and alerting, and available as a rich source of coded data for research. An important limitation, however, is that this type of resource would be expensive and difficult to port directly to other institutions, thus limiting its generalizability. The emergence of generative AI, and in particular large language models, makes it conceivable that some of these limitations might be reduced in the near future; for example, large language models might be used to perform a significant portion of the concept extraction task, turning clinical free text into sets of terms which might then be mapped to coded terminologies (such as the UMLS). This possibility is still largely hypothetical and will need to be investigated to evaluate whether it is feasible, performant, and economically viable.

It is also worth noting that in addition to the population-level analysis features offered by the Word Cloud as described in our research collaboration for PheWAS analysis, the CKM also uses its capability of providing summary views of individual patient charts for other projects, such as our synthesized evidence provision services [14,26]. In response to providers’ complex clinical questions, information scientists consult the visual display of the Word Cloud to gain a holistic understanding of each patient’s comorbidities, medications, and other prominent clinical history. This greatly facilitates our ability to generate tailored syntheses of the published evidence that are personalized to each specific patient case [26]. Additional applications of the Word Cloud and other AI tools are also under exploration at our center, including the use of AI for scaling the maintenance of evidence syntheses over time [27-29]. Through both of these approaches—leveraging the Word Cloud NLP for population-level concept analysis and individual patient-level assessment—the CKM achieves the rapid knowledge acquisition strategy critical for informing clinical health care and research at our institution.

## Abbreviations

AI: artificial intelligence
CKM: Center for Knowledge Management
EHR: electronic health record
NLP: natural language processing
PheWAS: phenome-wide association study
UMLS: Unified Medical Language System
VUMC: Vanderbilt University Medical Center
